# Measuring Spatial Patterns of Health Care Facilities and Their Relationships with Hypertension Inpatients in a Network-Constrained Urban System

**DOI:** 10.3390/ijerph16173204

**Published:** 2019-09-02

**Authors:** Zhensheng Wang, Ke Nie

**Affiliations:** 1 Key Laboratory of Urban Land Resources Monitoring and Simulation, Ministry of Land and Resources of China, Shenzhen 518034, China; 2 Key Laboratory for Geo-Environmental Monitoring of Coastal Zone of the Ministry of Natural Resources & Guangdong Key Laboratory of Urban Informatics & Shenzhen Key Laboratory of Spatial Smart Sensing and Services, Shenzhen University, Shenzhen 518060, China; 3 College of Civil and Transportation Engineering, Shenzhen University, Shenzhen 518060, China; 4 Research Institute for Smart Cities, School of Architecture and Urban Planning, Shenzhen University, Shenzhen 518060, China

**Keywords:** health care facilities, hypertensive inpatient, health care accessibility, network-constrained urban system

## Abstract

There is evidence of a strong correlation between inequality in health care access and disparities in chronic health conditions. Equal access to health care is an important indicator for overall population health, and the urban road network has a significant influence on the spatial distribution of urban service facilities. In this study, the network kernel density estimation was applied to detect the hot spots of health care service along the road network of Shenzhen, and we further explored the influences of population and road density on the aggregate intensity distributions at the community level, using spatial stratified heterogeneity analyses. Then, we measured the spatial clustering patterns of health care facilities in each of the ten districts of Shenzhen using the network K-function, and the interrelationships between health care facilities and hypertension patients. The results can be used to examine the reasonability of the existing health care system, which would be valuable for developing more effective prevention, control, and treatment of chronic health conditions. Further research should consider the influence of nonspatial factors on health care service access.

## 1. Introduction

China has experienced a dramatic demographic change and epidemiologic transition in the past three decades [1]. Although the urbanization rate reached 53.73% in 2013, a large number of migrants, who do not have local household registration due to China’s ‘Hukou’ policy, are not entitled to social welfare from the local government, including access to labour employment and health care insurance schemes [2]. This has caused their health care, housing, and education needs to go unmet, inevitably leading to a variety of social conflicts and inequitable problems [3]. Therefore, it is essential to consider quality improvements and the implementation of a high-efficiency health care system when developing the sustainability of health care reform in China.

In modern cities, health care facilities are part of the public infrastructure, providing beneficial functions for urban residents, including pre-hospital emergency medical care, primary care services, and community health care services. Based on China’s hierarchical administrative structure, the health care system is a service network composed of clinics, community health centers (CHCs), and hospitals, including primary hospitals, secondary hospitals and tertiary hospitals [4]. This multi-level system provides daily health care services to the vast majority of urban residents. However, a highly bureaucratic and centralized health care delivery system, with resources consolidated in large hospitals, was formulated due to the planned economy before the 1980s. Public hospitals provide a large proportion of all outpatient and inpatient services. Therefore, residents tend to seek health care services at large public hospitals, even for mild health problems, rather than local primary health care facilities [5], which could lead to a low quality of health care services received by the residents and a waste of medical resources [6]. Furthermore, due to competition or collaboration, there may be significant interrelationships among different types of health care facilities, such as hospitals and clinics [7], which needs to be examined. Improvements in the overall efficiency of the health care system are essential for equity in health and health care in China. Within the concept of health equity, equal access to health care is a critical indicator for overall population health. Therefore, exploring the spatial distribution patterns of health care facilities is essential to optimize the spatial allocation of health care resources, and mitigate disparities in health care service.

There is evidence of a strong correlation between inequality in health care access and disparities in various health outcomes, including low birth weight, maternal mortality, circulatory disease, cardiovascular events, late-stage cancer diagnosis, and others. Hypertensive disease is regarded as the most common non-communicable disease (NCD) in China, affecting over 200 million patients [8], but hypertension awareness, treatment, and control rates are low [9]. Moreover, high blood pressure is a significant risk factor for cardiovascular disease (CVD) and other vascular diseases [10]. The treatment of hypertensive disease and its coexisting conditions requires a massive consumption of health care resources [11]. A lack of accessibility to health care facilities could result in an increasing lack of early diagnosis, control, and management of high blood pressure.

Health care accessibility refers to the ease or difficulty of reaching services from a given location [12,13], which can be considered an interface between potential users and health care resources [14]. Potential access focuses on the spatial distribution of health care services, while realized access refers to the actual use of services [12,15]. Many barriers impede the progression from potential to realized access, and spatial accessibility emphasizes the importance of spatial separation between supply and demand, and how they are connected in geographical space [13,16], which is generally accepted as a basis for optimizing the allocation of health care resources in urban areas [12,17]. Factors affecting health care spatial accessibility include the location of health care services, and how services will be reached [15]. When the spatial allocation of health care facilities and services can meet the residents’ needs, the supply can be considered adequate and accessibility as convenient. Due to the increasing availability of detailed geo-referenced data and improved analysis methods, geographic information system (GIS) technologies provide a unified framework for allowing inferences about the relationships among multi-source data in a geographical context [18,19].

In a GIS environment, health care facilities, just like many other geographical phenomena such as car crashes on a road, residential locations, crimes and disease outbreak sites, can be abstracted as points for spatial analysis. Point pattern analysis (PPA) has been widely applied to investigate the global or local spatial distribution patterns of point events [20,21]. In traditional analysis methods, it is generally assumed that spatial events can be located stochastically on a plane, and the spatial association between event locations is analysed using the Euclidean (or planar) distance [22,23,24]. However, this assumption is not appropriate when a spatial phenomenon is apparently constrained to a subset of geographical space, such as a road network. Urban road network plays a vital role in influencing the formation of human activities. There are many events, such as car crashes on roads, health care services alongside streets, and street crimes, which are strongly restricted by the road network and which can therefore be termed network-constrained events [25,26,27]. These events can be categorized into alongside network and on-network events. A large proportion of urban service facilities can be considered alongside-network events. Therefore, Euclidean-based methods, which are designed for events occurring on a continuous plane, may not be suitable for characterizing network-constrained point events [25,28,29]. Furthermore, the distance to a health care provider along the road network was recognized as a significant factor affecting the equality of health care accessibility [16,30]. In recent years, many researchers have made significant progress by extending planar spatial analysis methods to network-contrained point events, such as by using the network Kernel density estimation [28,31,32] and network K-function [26,33,34].

Distance-related inequality in health care service use is potentially a significant public health issue, and the interrelation between spatial accessibility to health care services and the disparities in chronic diseases is particularly interesting in China. Therefore, this paper aims to fulfill two research objectives in a highly urbanized area of China: Shenzhen. The first objective is to measure the spatial distribution characteristics of health care facilities using network-based analysis methods. The second objective is to explore the interrelationship between health care facilities and hypertension patients in the road network space. The next section of this paper describes the materials and methods. Section 3 presents the analysis results and discusses the findings. Section 4 concludes the study.

## 2. Materials and Methods

### 2.1. Study Area

**Figure 1:**
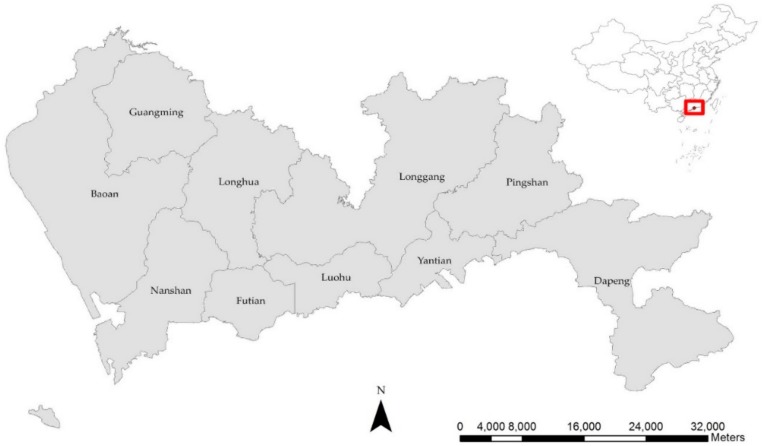
The location of Shenzhen and its administrative division.

Shenzhen is located in the south of Guangdong province, bordering Dongguan in the north, Huizhou in the north and northeast, Hong Kong across the Shenzhen River in the south, Lingdingyang and the Pearl River in the west, and Mirs Bay in the east (Figure 1). The study area is comprised of 10 districts, 57 sub-districts, and 648 communities (average 28,520 inhabitants, area 3.04 km^2^). Shenzhen has become a modern metropolis with well-developed secondary and tertiary industries, and is one of the fastest-growing cities in the world. By the end of 2014, after the reclamation of some coastal wetlands, Shenzhen had a total population of approximately 20 million and the municipality covered an area of 1991.64 km^2^, including urban and rural areas [35]. The total annual expenditure for medical and health care in 2017 was 24.4 billion RMB, with a yearly increase of 32% from 2013. The per capita consumption expenditure of permanent households for health care and medical services in 2017 was 1154 RMB [35]. Shenzhen has a unique population structure, as a large proportion of its total population is recognized as part of migrant or floating populations, which leads to obstacles in implementing policies to improve the public health care system [2,36]. 

### 2.2. Data Sources

**Figure 2:**
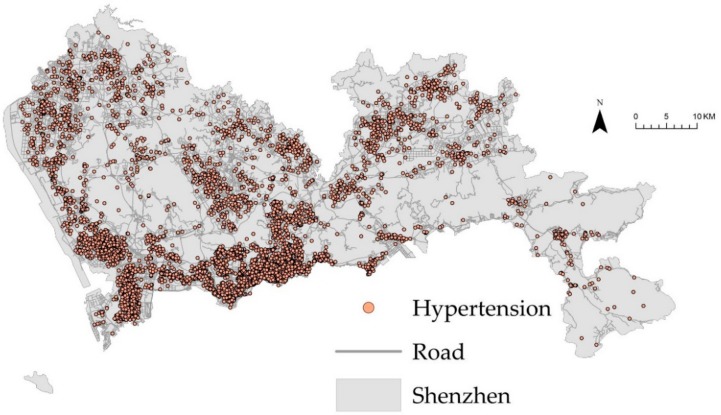
Point map of hypertension patients and the road network in the study area.

Data on hypertension inpatients were provided by Shenzhen Center for Health Information, with 10,395 cases recorded in 2013. We obtained data for primary hypertension (ICD-10 codes I10, the 10th revision of the International Statistical Classification of Diseases and Related Health Problems), hypertension heart disease (ICD-10 I11), hypertension renal disease (ICD-10 I12), hypertension heart and renal disease (ICD-10 I13), and secondary hypertension (ICD-10 I15). The hypertension patients were mapped to point events using geocoding tools [37] based on their home addresses, recording the structural hierarchy of China’s administrative divisions based on street number, street name, sub-district name, and district name. Figure 2 shows the spatial distribution of hypertension cases (as solid red circles) in the study area.

**Figure 3:**
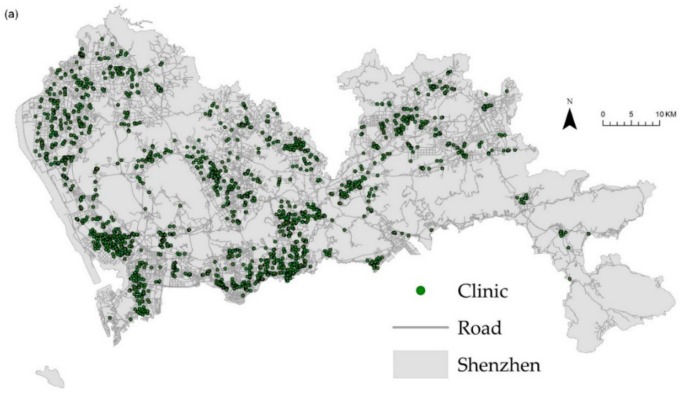

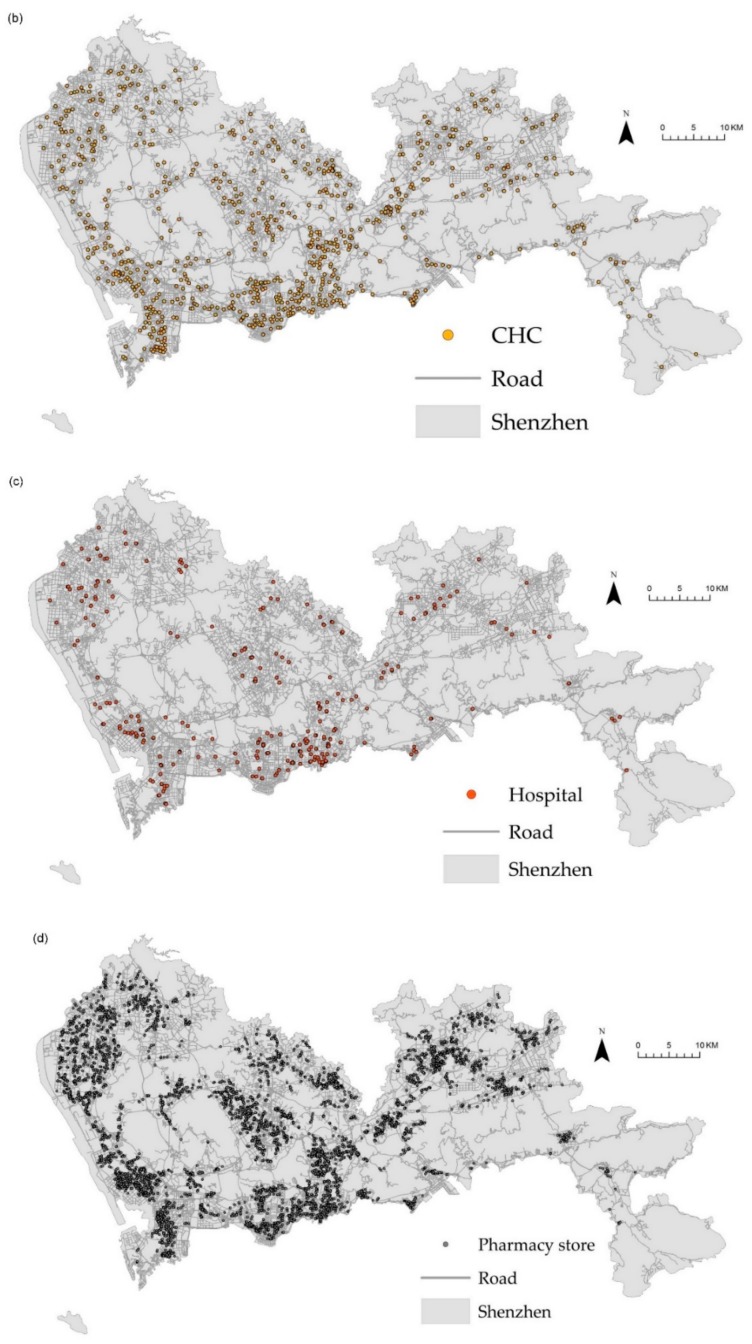
Distribution of health care facilities including: (**a**) clinics; (**b**) community health centers (CHCs); (**c**) hospitals; and (**d**) pharmacy stores.

Data on the health care facilities were obtained from the Urban Planning, Land and Resources Commission of Shenzhen Municipality, including 344 hospitals, 751 CHCs, 2035 clinics, and 5519 pharmacy stores that were abstracted as point locations for spatial analysis. We also included the bed numbers of the hospitals to represent their capacity to accommodate patients. Figure 3 shows the spatial distribution of health care facilities and pharmacy stores, along with 7885 km of the road network.

### 2.3. Network Kernel Density Estimation

Network kernel density estimation (NetKDE) is a nonparametric method that aims to examine the first-order properties of spatial data [32], which estimates the intensity of point events in a network space using a kernel function. NetKDE applies a bandwidth that is represented by the shortest-path distance along the network, rather than a planar Euclidean distance measure:

(1) $$\lambda (s)=\sum_{i=1}^{n}\frac{1}{r}k(\frac{d_{is}}{r})$$

where $\lambda \left(s\right)$ denotes the KDE density at location s; i represents the point event, where i=1,…,n; r is the bandwidth of the NetKDE and only point events within r are calculated; $d_{is}$ is the distance between the estimation point s and the observation point i; and k() is a kernel function of the ratio between $d_{is}$ and r. Previous studies have indicated that the choice of the kernel function is less important than the choice of search bandwidth [28,38]. Thus, we apply the most commonly used kernel function, the Gaussian function:

(2) $$k(\frac{d_{is}}{r})=\left\{\begin{array}{c} \frac{1}{\sqrt{2\pi }}\times \exp(-\frac{d_{is}^{2}}{2r^{2}})\times w_{i},0<d_{is}\leq r\\ 0,d_{is}>r \end{array}\right.$$

where $w_{i}$ is the non-spatial factor of the point event i. In this study, the bed numbers of health care facilities are used to model the importance of the point event i.

### 2.4. Network K-Function

The network K-function method uses the shortest-path distance to test the complete spatial randomness (CSR) hypothesis that points are independently and identically distributed according to the uniform distribution over the network, or points follow the homogeneous binomial point process on the bounded network [33]. We applied the network auto K-function and network cross K-function method in this study [39]. The former deals with a single type of point events and the latter focus on two sets of points of different types (e.g., residential locations and schools), to uncover the spatial interrelationships between two-point sets.

For a set of points, $P=\left\{p_{1},\ldots ,p_{n}\right\}$ located over a network, the network K-function at point $p_{i}$ is formulated with the number of points within distance t from a specific point $p_{i}$ to the other points in P, which is defined as follows:

(3) $$K(t|p_{i})=\frac{1}{\rho }n(t|p_{i})$$

where $n(t|p_{i})$ is the number of points that are within the shortest-path distance t from point $\;p_{i}$; $\rho =\left(n-1\right)/\left|\tilde{S}\right|$ is the density of points on the network, and $\left|\tilde{S}\right|$ is the total segment length of the network. To test the CSR hypothesis, the Monte Carlo simulation is widely used to quantify the distribution pattern of network-constrained point events [27,33,40]. The differences between the observed K-function values and the CSR point pattern test could indicate that whether the network events are uniformly and independently distributed. If the observed K-function values $K\left(t\right)$ exceed the upper CSR bound, the distribution of the point set P is a clustering pattern; if the observed K-function values $K\left(t\right)$ are below the lower CSR bound, the point set P shows a dispersion distribution; if the values of $K\left(t\right)$ are in the range of the envelope curve, the point set P is in a random distribution. The cross K-function explores the spatial interrelationship of two types of points.

### 2.5. Spatial Stratified Heterogeneity Analyses

The geographical detector method [41] is a spatial analysis method for measuring spatial stratified heterogeneity [42,43]. It was applied in this study to examine whether multiple variables (i.e., population density and road density) independently or dependently affect the aggregate intensity of the health care facilities along the road network at the community level. The two variables, namely population densities and road lengths, were divided into five ordinal categories using Jenk’s optimization data classification, which reduces the within-group variance while maximizing the between-group variance. The effects of the two variables on the intensity of the health care facilities may be stronger or weaker after the interaction (Table 1). Formally, a study area is composed of N units and is stratified into h=1,2,…,L stratum; stratum h is composed of $N_{h}$ units; $Y_{i}$ and $Y_{hi}$ indicate the value of unit i in the population and in stratum h, respectively; ${\bar{Y}}_{h}$ and $\bar{Y}$ denote the mean value of the stratum and the population, respectively; and $\sigma_{h}^{2}$ and $\sigma^{2}$ denotes the stratum variance and the population variance, respectively. The q-statistic, the measure of spatial stratified heterogeneity, is calculated as follows:

(4) $$q=1-\frac{\sum_{h=1}^{L}\sum_{i=1}^{N_{h}}{\left(Y_{hi}-{\bar{Y}}_{h}\right)}^{2}}{{\sum_{i=1}^{N}\left(Y_{i}-\bar{Y}\right)}^{2}}=1-\frac{\sum_{h=1}^{L}N_{h}\sigma_{h}^{2}}{N\sigma^{2}}$$

The value of the q-statistic is between $\left[0,1\right]$ (0 if there is no stratified heterogeneity, and 1 if the population is fully stratified), and it increases as the strength of the stratified heterogeneity increases.

## 3. Results and Discussion

### 3.1. NetKDE Analysis of Health Care Facilities

We considered the influence of non-spatial factors of health care facilities in the analysis. Here, the intensity of hospitals was weighted by the bed numbers of each health care facility, representing the importance of the point event. According to the service capacity standards of CHCs and Shenzhen health statistics, CHCs, clinics, and pharmacy stores were assigned with weights of 20, 10, and 1, respectively. The computation of NetKDE was implemented in the ESRI ArcGIS 10.5 environment, using Microsoft Visual C# 2016. The choice of search bandwidth is an essential issue in KDE; a larger bandwidth may lead to unexpected generalizations, and a smaller bandwidth could over-emphasize local variation. Previous studies suggested that a 300 m bandwidth was suitable for the study of activities of urban residents [31,44], which is not applicable in our case. The reason for is that our study region has a larger spatial extent, with 7885 km of the road network, and those small bandwidths could cause a “spiky” pattern phenomenon. After several rounds of experiments, a 1000 m bandwidth was chosen to calculate the NetKDE of the hospital facilities. Because local clinics and CHCs serve a smaller area than hospitals, a 500 m bandwidth was therefore used. The cell size for the output NetKDE raster dataset was set as 100 m. 

In the visualization of density surface, we applied Jenks classification based on the statistical characteristics of the NetKDE values. We further extracted the isolines of different classification numbers and then calculated those weight means of the isolines. After several rounds of experiments with different numbers of classification, we set the number of classes to five because there is no significant variation in the weight means of different numbers of classification.

**Figure 4:**
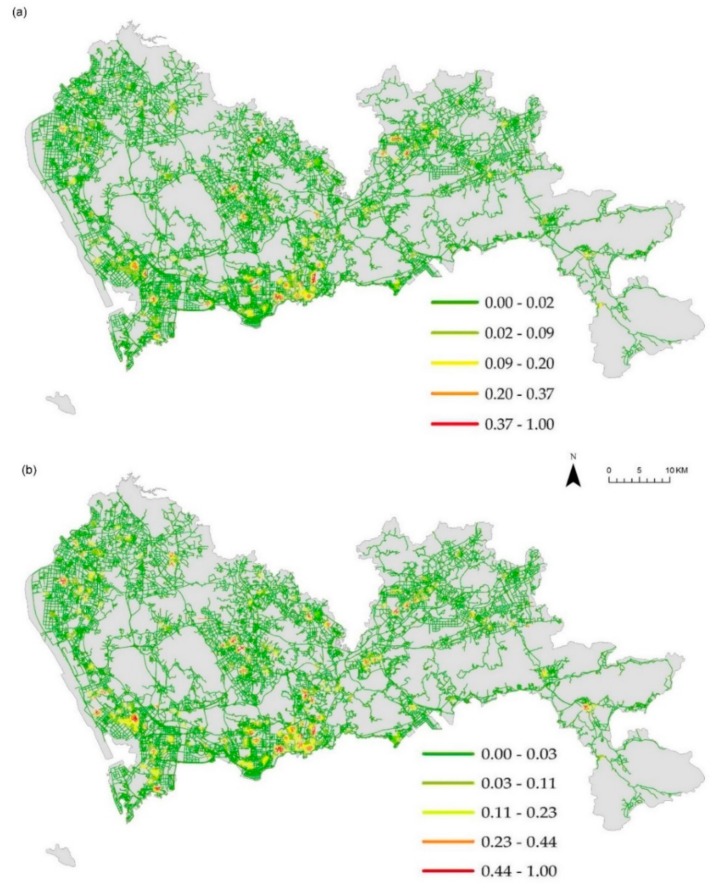
The intensity distribution of hospital facilities: (**a**) weighted NetKDE values using bed numbers; (**b**) unweighted NetKDE values.

**Figure 5:**
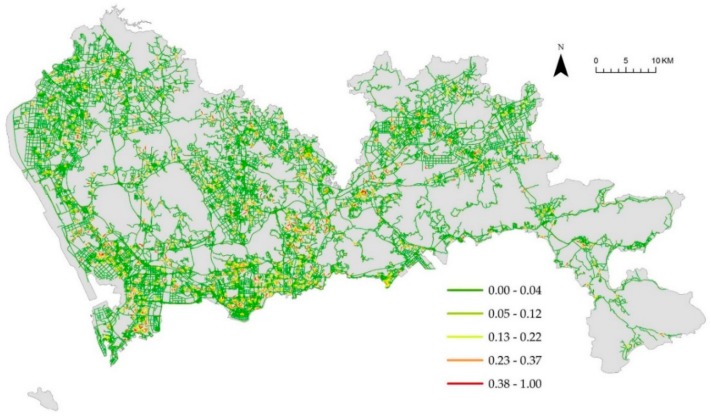
The intensity distribution of CHCs using the weighted NetKDE method.

**Figure 6:**
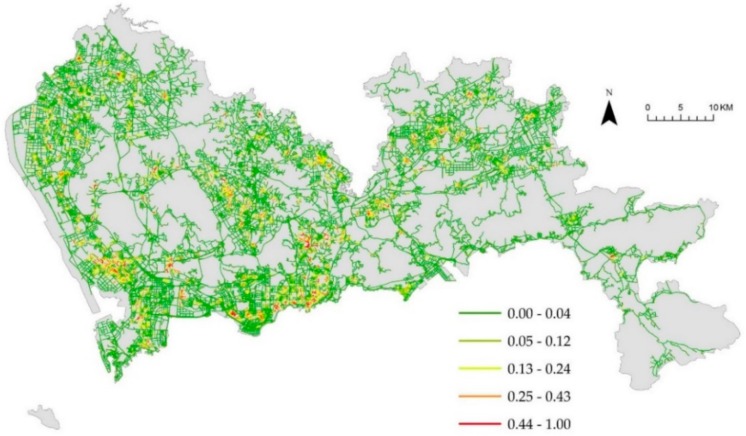
The intensity distribution of clinics using the weighted NetKDE method.

**Figure 7:**
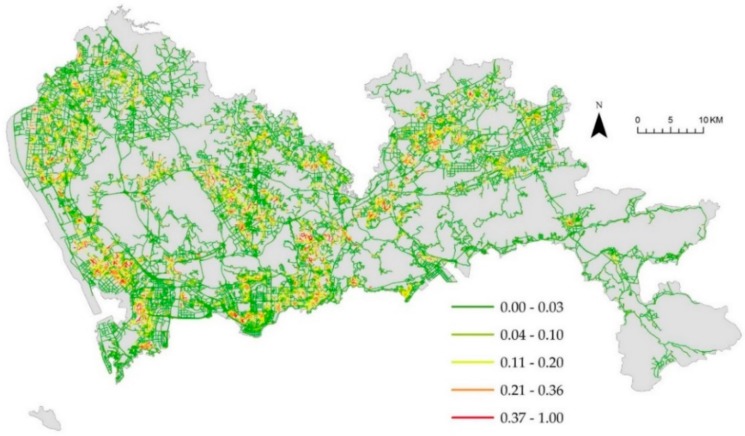
The intensity distribution of pharmacy stores using the weighted NetKDE method.

Figure 4a denotes the weighted NetKDE values of the hospital facilities based on their capacity to accommodate patients. We also computed the unweighted NetKDE values of hospitals, as shown in Figure 4b. The NetKDE method detects hot spots by calculating the density values of hospital facilities along the road network. By comparing Figure 4a,b, we can see that the bed numbers of the hospitals have a significant influence on the density values of hospital facilities. There are 14 tertiary hospitals (over 1000 bed numbers) located in Futian and Luohu district. Thus, the higher NetKDE values of hospital facilities are mainly concentrated in southern Shenzhen, and there is a significant difference in density values between the downtown and suburban area. Because there are many smaller hospitals with fewer bed numbers than large hospitals, the density values in the southwest may be overestimated using the unweighted NetKDE method that depends on the density of point events along the road network. Figure 5, Figure 6 and Figure 7 show the weighted NetKDE values of CHCs, clinics, and pharmacy stores, respectively. The intensity values of CHCs, clinics, and pharmacy stores along networks exhibit a significant distribution pattern of spatial heterogeneity. The higher NetKDE values of CHCs and clinics are mainly located in areas with high road density. Because pharmacy stores usually have a close connection with the health care needs of urban residents, the intensity distribution of pharmacy stores is highly correlated with the population distribution characteristic. 

The sum of the NetKDE values of the health care facilities was aggregated at the community level. Spatial stratified heterogeneity analyses were further applied to explore whether population density and road density simultaneously affect the aggregate intensity of health care facilities, as estimated by NetKDE methods. The average population density and road density per square kilometer was 29,132.10 and 7350.59 m. Table 1 summarizes the interaction effects between population densities and road density for different kinds of health care facilities at the community level. The q-statistics of the population density and road density were 0.022 and 0.024 for aggregate NetKDE values of health care facilities, respectively, using the geographical detector method [41]. The spatial stratified heterogeneity analysis indicated significant associations (p<0.05) between the aggregate NetKDE values and the population densities and the road densities, respectively, at the community level. The total intensities were further elevated by interaction effects between the population and road densities. 

### 3.2. Spatial Cluster Pattern Analysis

**Figure 8:**
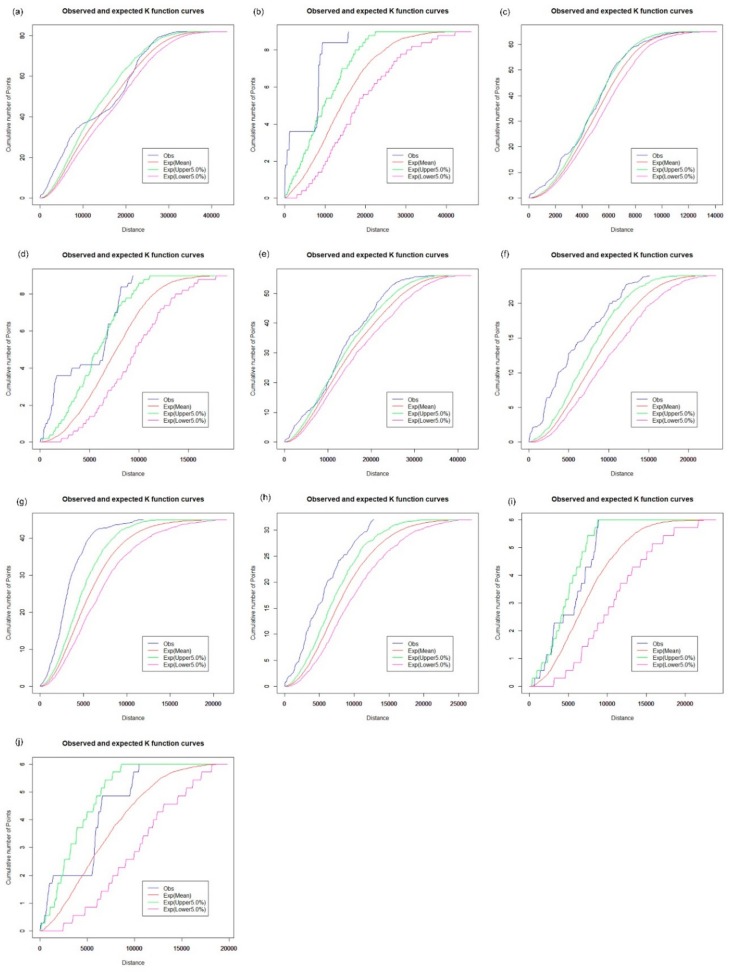
Network auto K-function analysis of hospitals. (**a**) Baoan; (**b**) Dapeng; (**c**) Futian; (**d**) Guangming; (**e**) Longgang; (**f**) Longhua; (**g**) Luohu; (**h**) Nanshan; (**i**) Pingshan; (**j**) Yantian.

**Figure 9:**
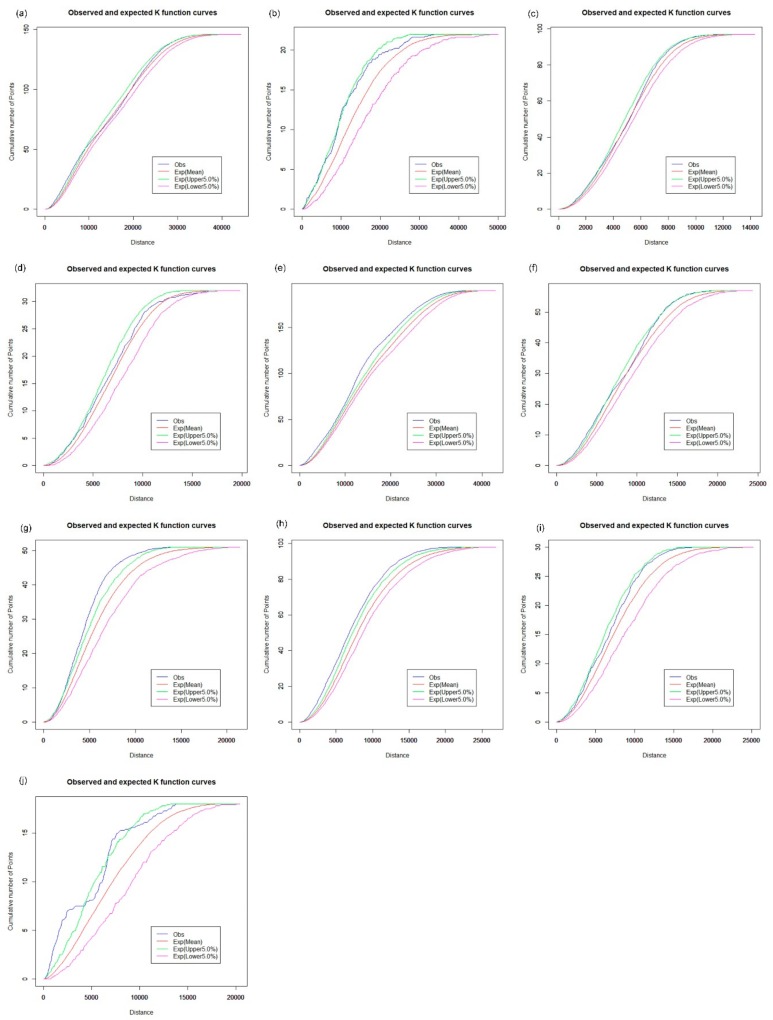
Network auto K-function analysis of CHCs. (**a**) Baoan; (**b**) Dapeng; (**c**) Futian; (**d**) Guangming; (**e**) Longgang; (**f**) Longhua; (**g**) Luohu; (**h**) Nanshan; (**i**) Pingshan; (**j**) Yantian.

**Figure 10:**
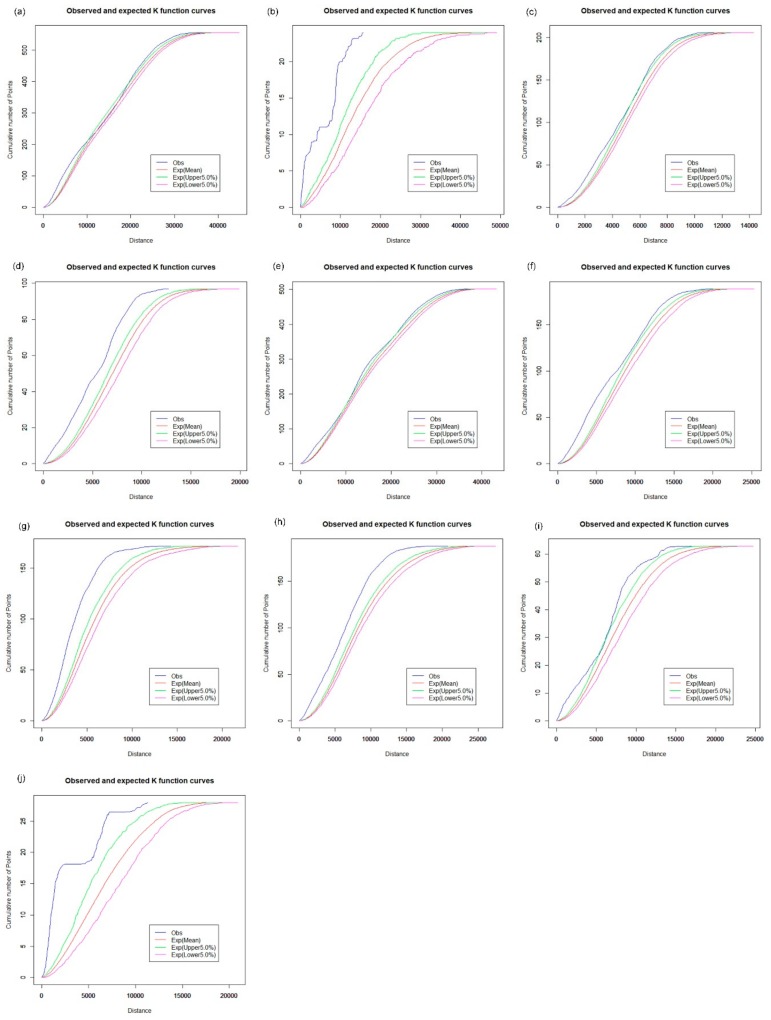
Network auto K-function analysis of clinics. (**a**) Baoan; (**b**) Dapeng; (**c**) Futian; (**d**) Guangming; (**e**) Longgang; (**f**) Longhua; (**g**) Luohu; (**h**) Nanshan; (**i**) Pingshan; (**j**) Yantian.

**Figure 11:**
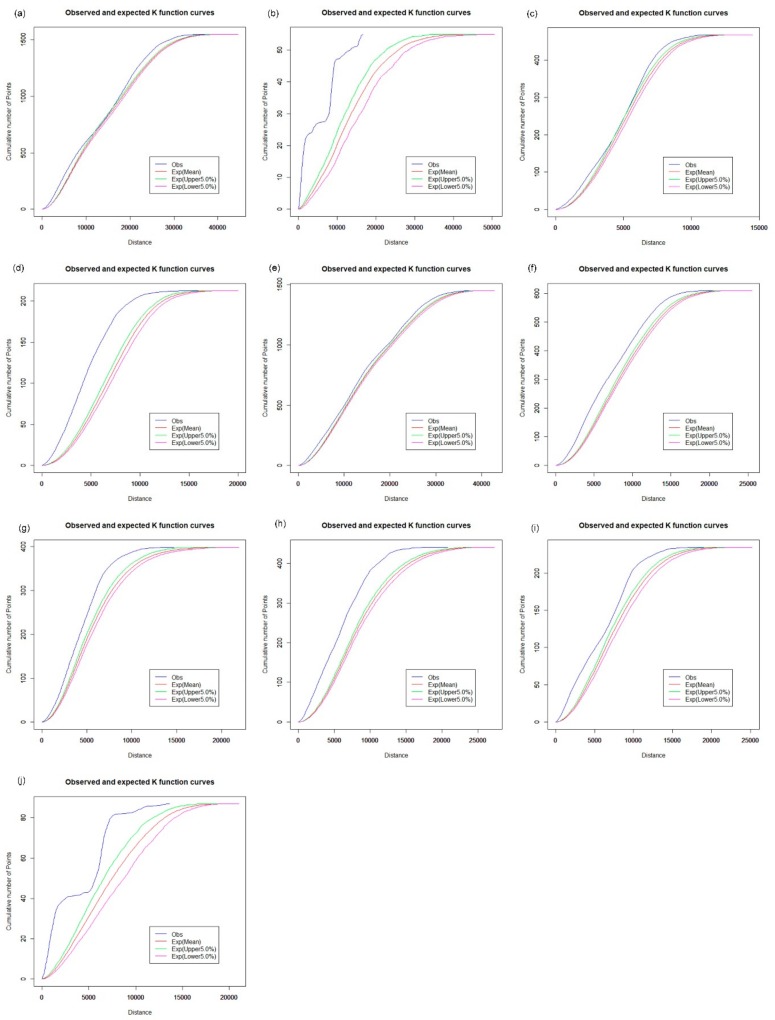
Network auto K-function analysis of pharmacy stores. (**a**) Baoan; (**b**) Dapeng; (**c**) Futian; (**d**) Guangming; (**e**) Longgang; (**f**) Longhua; (**g**) Luohu; (**h**) Nanshan; (**i**) Pingshan; (**j**) Yantian.

The Spatial Analysis along Networks (SANET) toolbox [27] was applied to calculate the network K-function values. We first explore the spatial cluster patterns of hospitals, CHCs, clinics, and pharmacy stores for each of the 10 districts using the network auto K-function method. The results are shown in Figure 8, Figure 9, Figure 10 and Figure 11, and were obtained from the R language window. The parameters “Exp (upper 5.0%)” and “Exp (lower 5.0%)” indicate the upper and the lower envelope curves of the Monte Carlo simulation with the significance level of 0.05. The parameter “Exp (Mean)” is the expectation of random distribution, and the parameter “obs” denotes the observed K-function values. The vertical axis indicates the cumulative number of point events; the horizontal axis is the distance range. For example, as shown in Figure 8a, the observed blue curve is above the upper envelope of the curves under the distance of 10,000 m, which indicates that we can reject the CSR hypothesis with a 0.95 confidence level. Hence, hospitals in Baoan tend to present a cluster pattern in that distance range. Figure 8c shows that the observed curve of the network K-function nearly coincides with the upper envelope curve under the CSR hypothesis, which indicates that the distribution of hospitals is even in the Futian district.

The results of the network-auto K-function indicate that hospitals tend to be in a clustered distribution in Baoan (Figure 8a), Dapeng (Figure 8b), Guangming (Figure 8d), Longgang (Figure 8e), Longhua (Figure 8f), Luohu (Figure 8g), and Nanshan (Figure 8h). Hospitals in Pingshan and Yantian show a significantly random pattern, as shown in Figure 8i,j, respectively, which indicates that there is an apparent imbalance in the health care services of hospital facilities across Shenzhen, in part because the majority of tertiary and secondary hospitals are mainly located in the downtown area. In terms of health care inequity, urban planners should strategically pay attention to the planning of large hospitals to satisfy the future needs of urban residents.

The spatial distribution of the CHCs exhibits a different pattern. For the analysis of the spatial cluster pattern of the CHCs, Figure 9 shows that the observed curve of the network K-function is between the upper and lower envelope curves. Hence, CHCs in Baoan tend to be in a random distribution in the street network space (Figure 9a), as well as in Dapeng (Figure 9b), Futian (Figure 9c), Guangming (Figure 9d), Longhua (Figure 9f) and Pingshan (Figure 9i). However, the observed curve is slightly beyond the upper envelope curve for CHCs in other districts, as shown in Figure 9e,g,h,j. The results indicate that the spatial distribution of CHCs is relatively balanced between the downtown area and the central urban districts in Shenzhen. CHCs are an essential part of the primary health care system and are designed to provide essential public health services, which is valuable for the control and prevention of chronic diseases. Many measures, such as adequate education and qualification of the workforce, financial subsidies, and incentives, need to be further addressed to improve the performance of CHCs. Because there is a close connection between the small clinics, pharmacy stores, and the daily needs of urban residents, the results of the network K-function show that the spatial distribution of the clinics has a clustered pattern across Shenzhen, which is similar to that of pharmacy stores, as shown in Figure 10 and Figure 11.

**Figure 12:**
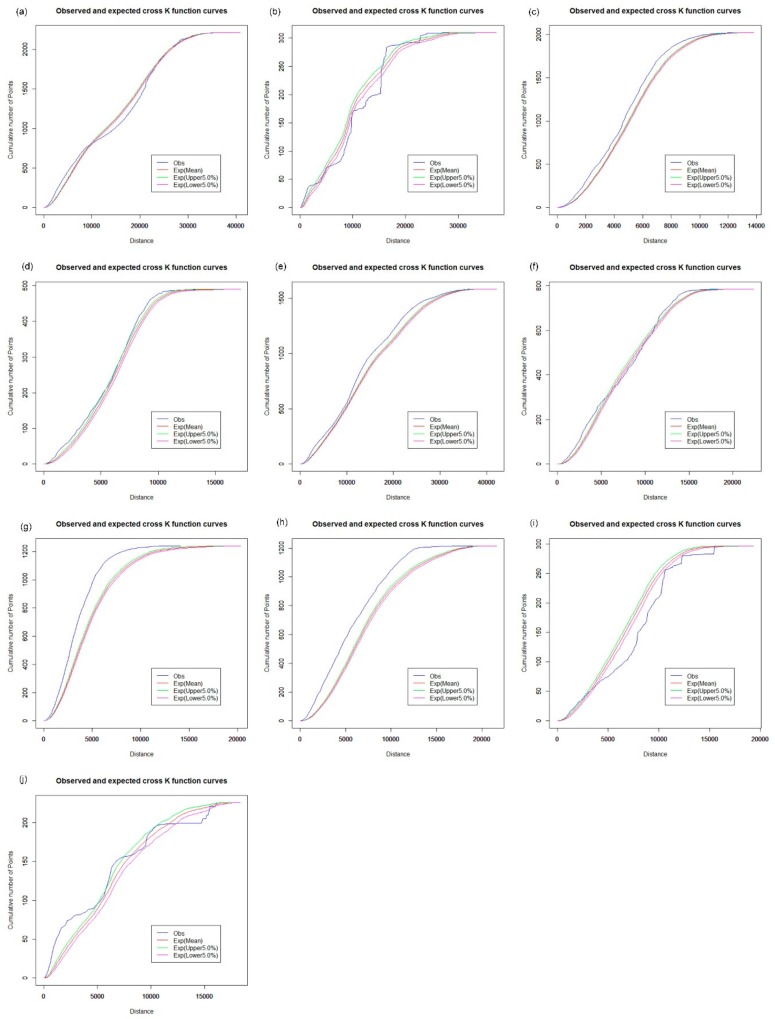
Network cross K-function analysis of hospitals and hypertension patients. (**a**) Baoan; (**b**) Dapeng; (**c**) Futian; (**d**) Guangming; (**e**) Longgang; (**f**) Longhua; (**g**) Luohu; (**h**) Nanshan; (**i**) Pingshan; (**j**) Yantian.

The network cross K-function was applied to analyze the relationship between hypertension patients and health care facilities across 10 districts of Shenzhen. The results were used to investigate whether the configuration of one type of point could influence the distribution of another kind of point event. We first examined the interrelationship between hospitals and hypertension patients in the road network space across the Shenzhen Municipality. Figure 12 shows that the interrelationship between hospitals and hypertension patients is significantly different across the 20 districts of Shenzhen, with a significance level of 5%. The observed values of the network cross K-function of hospitals and hypertension patients are above the upper envelope of the CSR hypothesis in Futian (Figure 12c), Longgang (Figure 12e), Luohu (Figure 12g) and Nanshan (Figure 12h), which indicates that there is a significant cluster for a spatial distribution relationship between the hospitals and hypertension patients along the road network in these areas. However, the observed curve approximates the envelope curves under the CSR hypothesis in Baoan (Figure 12a), Guangming (Figure 12d) and Longhua (Figure 12f); the observed curve is below the lower envelope in Pingshan (Figure 12i), and there is a complicated interrelationship between hospitals and hypertension patients in Dapeng (Figure 12b) and Yantian (Figure 12j).

**Figure 13:**
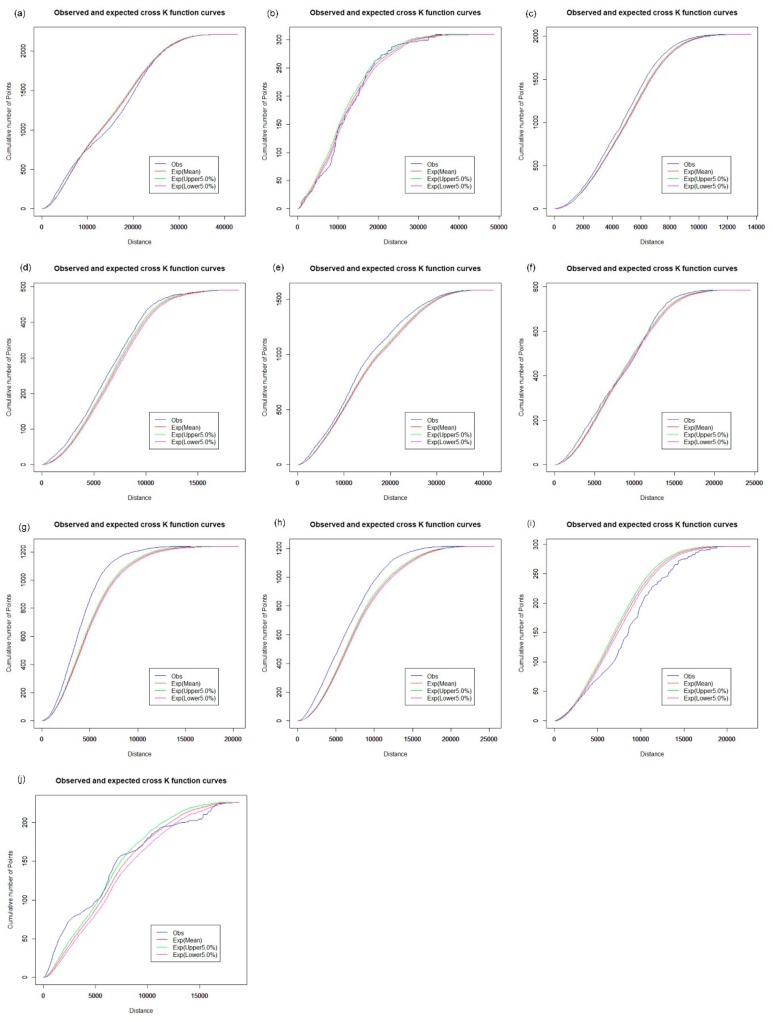
Network cross K-function analysis of CHCs and hypertension patients. (**a**) Baoan; (**b**) Dapeng; (**c**) Futian; (**d**) Guangming; (**e**) Longgang; (**f**) Longhua; (**g**) Luohu; (**h**) Nanshan; (**i**) Pingshan; (**j**) Yantian.

**Figure 14:**
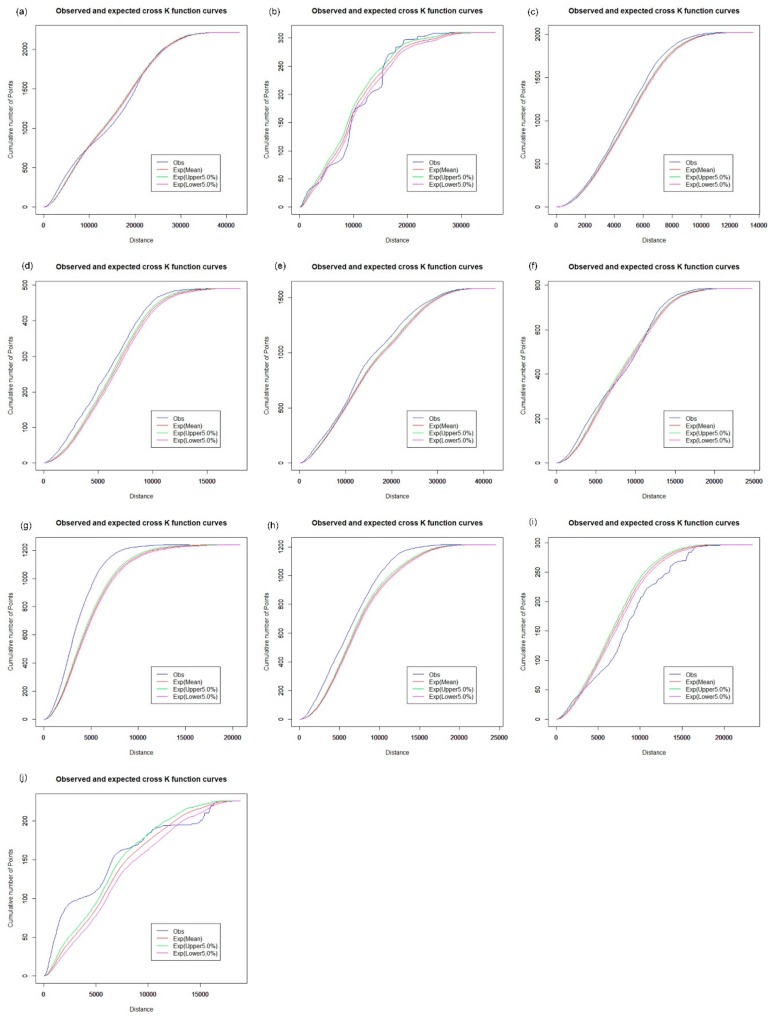
Network cross K-function analysis of clinics and hypertension patients. (**a**) Baoan; (**b**) Dapeng; (**c**) Futian; (**d**) Guangming; (**e**) Longgang; (**f**) Longhua; (**g**) Luohu; (**h**) Nanshan; (**i**) Pingshan; (**j**) Yantian.

**Figure 15:**
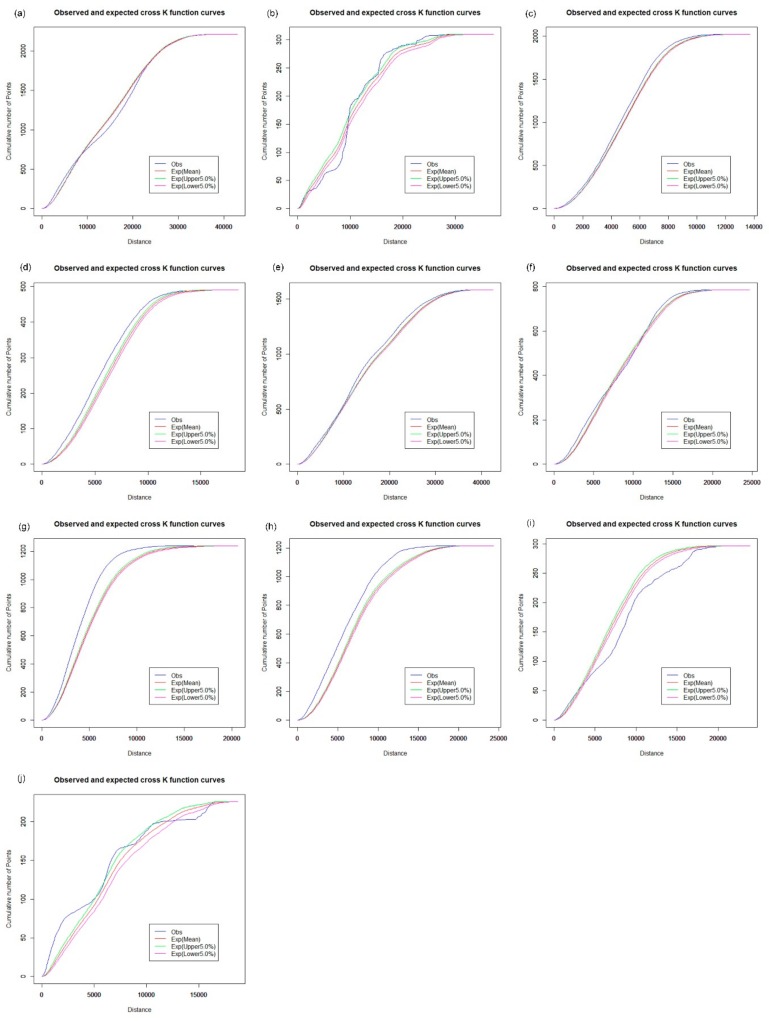
Network cross K-function analysis of pharmacy stores and hypertension patients. (**a**) Baoan; (**b**) Dapeng; (**c**) Futian; (**d**) Guangming; (**e**) Longgang; (**f**) Longhua; (**g**) Luohu; (**h**) Nanshan; (**i**) Pingshan; (**j**) Yantian.

We further examined the effects of CHCs, clinics, and pharmacy stores on the spatial distribution characteristics of hypertension patients in the road network space, as shown in Figure 13, Figure 14 and Figure 15, respectively. In Figure 13, the interrelationship between CHCs and hypertension patients exhibits a pattern which is similar to that of hospitals and hypertension patients in the road network space of Shenzhen (Figure 12), except in Dapeng and Guangming. Figure 13b shows that the observed value of the network cross K-function for CHCs and hypertension patients approximates the mean value of the envelope under the CSR hypothesis with a significance level of 5%. That is, compared with the CHCs, the distribution of hypertension patients is random in Dapeng. In Figure 13d, the observed curve is above the upper envelope under the CSR hypothesis, which demonstrates that hypertension patients tend to cluster around the CHCs in Guangming. We found a similar spatial distribution relationship between hypertension patients and clinics and pharmacy stores, respectively, based on the results of the network cross K-function, as shown in Figure 14 and Figure 15. We found a significant clustering relationship in Futian (Figure 14c and Figure 15c), Guangming (Figure 14d and Figure 15d), Longgang (Figure 14e and Figure 15e), Luohu (Figure 14g and Figure 15g) and Nanshan (Figure 14h and Figure 15h).The results of the network cross K-function indicate that the interrelationship between the health care facilities and hypertension patients shows a significant cluster pattern in Futian, Longgang, Luohu, and Nanshan. Thus, there is clear evidence of spatial inequality in health care accessibility for hypertension patients in Shenzhen. Compared with other health care facilities, the observed curve of the network cross K-function for hospitals and hypertension patients is higher, above the upper envelope under the CSR hypothesis, in Futian, Longgang, Luohu, and Nanshan. In other words, hypertension patients are located closer to hospitals along the road network in these areas. Although it may be convenient for hypertension patients to seek health care services from hospitals in these areas, the high-rank hospitals are usually overburdened because the public has greater trust in large public hospitals over local health clinics and CHCs, creating significant systemic inefficiencies. Furthermore, the distribution of hypertension patients is also correlated with the locations of the CHCs, clinics, and pharmacy stores. Thus, it is necessary to upgrade infrastructure, information, and communication technology in primary health care facilities, and expand the essential drug list (EDL) in pharmacy stores and CHCs.

## 4. Conclusions

This study analyzed the spatial distribution characteristics of health care facilities in the road network space and their relationship with hypertension patients of Shenzhen using network-based spatial analysis. NetKDE was applied to detect health care service hot spots, and weighting of different types of health care facilities could be useful to obtain a comprehensive understanding of the intensity distribution of health care facilities. We further explored the influences of population density and road density on the aggregate intensity of different types of health care facilities at the community level, by using spatial stratified heterogeneity analyses. Then, the network K-function method was applied to measure the spatial clustering patterns in each of the 10 districts of Shenzhen along the road network. The spatial distribution of health care facilities displays distinct patterns in the 10 districts of Shenzhen, as well as in the interrelationships between health care providers and hypertension patients. Although the number of hospitals is relatively small, there is a stronger clustering pattern relationship between hospitals and hypertension inpatients along the road network. Due to the limitations of the data, directions of hypertension inpatient’s travel are not considered in this study and road segments are treated equally without being further distinguished. However, road segments are different in their traffic flow capacity, directionality, and so on, which may have an impact on the directions of travel of hypertension inpatients, subsequently affecting the density estimation of health care facilities and their interrelationships with hypertension inpatients. When the data becomes available, it may be beneficial to incorporate them into the analyses.

In China, public hospitals dominate health care delivery, and patients can visit hospitals directly for all health care needs. This may lead to increasingly long lines and waiting times, which in turn, increase costs and decrease access to health care even though the hospitals are located close to residential areas. Strengthening primary health care could be a cost-effective way to detect, diagnose, treat, and manage hypertension patients, to alleviate the burden on higher-level health services and mitigate medical costs to both individuals and the health systems. However, given the difficulty in collecting data at an individual level, we cannot confidently assert that strengthened primary health care services would affect the health-seeking behavior of hypertension individuals. Nevertheless, our findings confirm the importance of primary health care facilities, which require long-term commitment from the department of urban planning and public health [8,45]. 

In this study, we investigated the interrelationship between hypertension patients and health care facilities in the road network space. However, the use of health care services is also influenced by nonspatial factors that include many demographic and socioeconomic variables (such as income, age, sex, and race), and which also interact with spatial accessibility [13]. It is necessary to integrate nonspatial factors at both the individual and neighborhood levels with spatial accessibility in a unified measure. Hence, more advanced statistical models such as the Bayesian hierarchical model are needed to account for the small population problem, spatial autocorrelation, and the multilevel structure of the data in further research.

## Figures and Tables

**Table 1:** Interaction between population densities and road densities in contributing to aggregate network kernel density estimation (NetKDE) values at the community level.

Types	Interaction Detector C = A ∩ B ^1^		Linear Combination A + B	Interpretation
Hospital	population ∩ road = 0.086	<	0.101 = population (0.037) + road (0.064)	↑ ^1^
CHC	population ∩ road = 0.046	>	0.018 = population (0.009) + road(0.009)	⇑ ^2^
Clinic	population ∩ road = 0.086	>	0.056 = population (0.034) + road (0.022)	⇑
Pharmacy	population ∩ road = 0.098	>	0.043 = population (0.020) + road(0.023)	⇑
Aggregate value	population ∩ road = 0.087	>	0.046 = population (0.022) + road (0.024)	⇑

^1^ A and B indicate two different predictors; ^2^ A ⇑  B denotes nonlinear enhancement of A and B when C > A + B; ^3^ A ↑  B means A and B enhance each other when C > A, B.
